# Lung organoids, useful tools for investigating epithelial repair after lung injury

**DOI:** 10.1186/s13287-021-02172-5

**Published:** 2021-01-30

**Authors:** Jing Kong, Shiyuan Wen, Wenjing Cao, Peng Yue, Xin Xu, Yu Zhang, Lisha Luo, Taigui Chen, Lianbao Li, Feng Wang, Jian Tao, Guozhong Zhou, Suyi Luo, Aihua Liu, Fukai Bao

**Affiliations:** 1 The Institute for Tropical Medicine, Kunming Medical University, Kunming, 650500 Yunnan China; 2 Department of Biochemistry and Molecular Biology, Kunming Medical University, Kunming, 650500 Yunnan China; 3 The School of Medicine, Kunming University, Kunming, 650214 China; 4 Department of Microbiology and Immunology, Kunming Medical University, Kunming, 650500 China; 5 Yunnan Province Key Laboratory of Children’s Major Diseases Research, The Children’s Hospital of Kunming, Kunming Medical University, Kunming, 650030 China

**Keywords:** Stem cell, Lung organoid, Lung injury, Epithelial repair

## Abstract

Organoids are derived from stem cells or organ-specific progenitors. They display structures and functions consistent with organs in vivo. Multiple types of organoids, including lung organoids, can be generated. Organoids are applied widely in development, disease modelling, regenerative medicine, and other multiple aspects. Various human pulmonary diseases caused by several factors can be induced and lead to different degrees of lung epithelial injury. Epithelial repair involves the participation of multiple cells and signalling pathways. Lung organoids provide an excellent platform to model injury to and repair of lungs. Here, we review the recent methods of cultivating lung organoids, applications of lung organoids in epithelial repair after injury, and understanding the mechanisms of epithelial repair investigated using lung organoids. By using lung organoids, we can discover the regulatory mechanisms related to the repair of lung epithelia. This strategy could provide new insights for more effective management of lung diseases and the development of new drugs.

## Introduction

Lung injury can be induced by various human pulmonary diseases, and it can damage the crucial physiological functions of the lung. For example, viruses such as the influenza virus and certain coronaviruses (including the recently emerged severe acute respiratory syndrome coronavirus (SARS-CoV)-2) induce strong inflammation, massive damage, and cell death to the lung epithelium [1, 2]. Post infection, the impaired epithelium may not be able resist infection by other pathogens, which may lead to worse damage and prolong the disease. Lifestyle habits such as smoking reduce epithelial integrity and cause dramatic epithelial remodelling that are relevant to chronic obstructive pulmonary disease (COPD) and lung cancer [3, 4]. Despite scientific advancement in terms of the diagnosis and treatment, the 5-year survival of lung cancer patients is ~ 15% [5].

Asthma leads to changes in the structure and function of the airway epithelium that affects human health on a large scale [6]. Idiopathic pulmonary fibrosis (IPF) is a serious disease characterised by abnormal lung epithelial cells and aggravated by a deficiency in repair of alveoli [7]. The prognosis of most patients with IPF is very poor, with a median survival of 3–5 years after the diagnosis [8].

These pulmonary diseases related to different areas of lung epithelial injury impair lung function, affect the quality of life of patients, and endanger human health. Investigating the response to lung epithelial injury can increase our insight into pulmonary diseases and aid their treatment. Lung organoids are useful tools for these investigations.

The “lung organoids” are one of many kinds of organoids. Like other organoids, the lung organoids can derive from stem cells or organ-specific progenitors through a self-organisation process [9–13]. The cultured process of lung organoids in vitro is strikingly different compared with that of a traditional cell culture. The lung organoids can simulate the developmental process of the lung, as well as recapitulate the three-dimensional (3D) organisational structure (such as alveolars, airways, and lung buds) and function of the lung in vitro [11–13]. They are used widely to study pulmonary diseases. In this review, we focus on the recent methods of deriving lung organoids, the applications of lung organoids in epithelial repair after injury, and understanding the mechanisms of epithelial repair that are investigated by lung organoids. We can discover the regulatory mechanisms related to the repair of lung epithelia, which could provide new insights for more effective management of lung injury and drug development.

## Lung organoids

### History of lung organoids

**Fig. 1:**
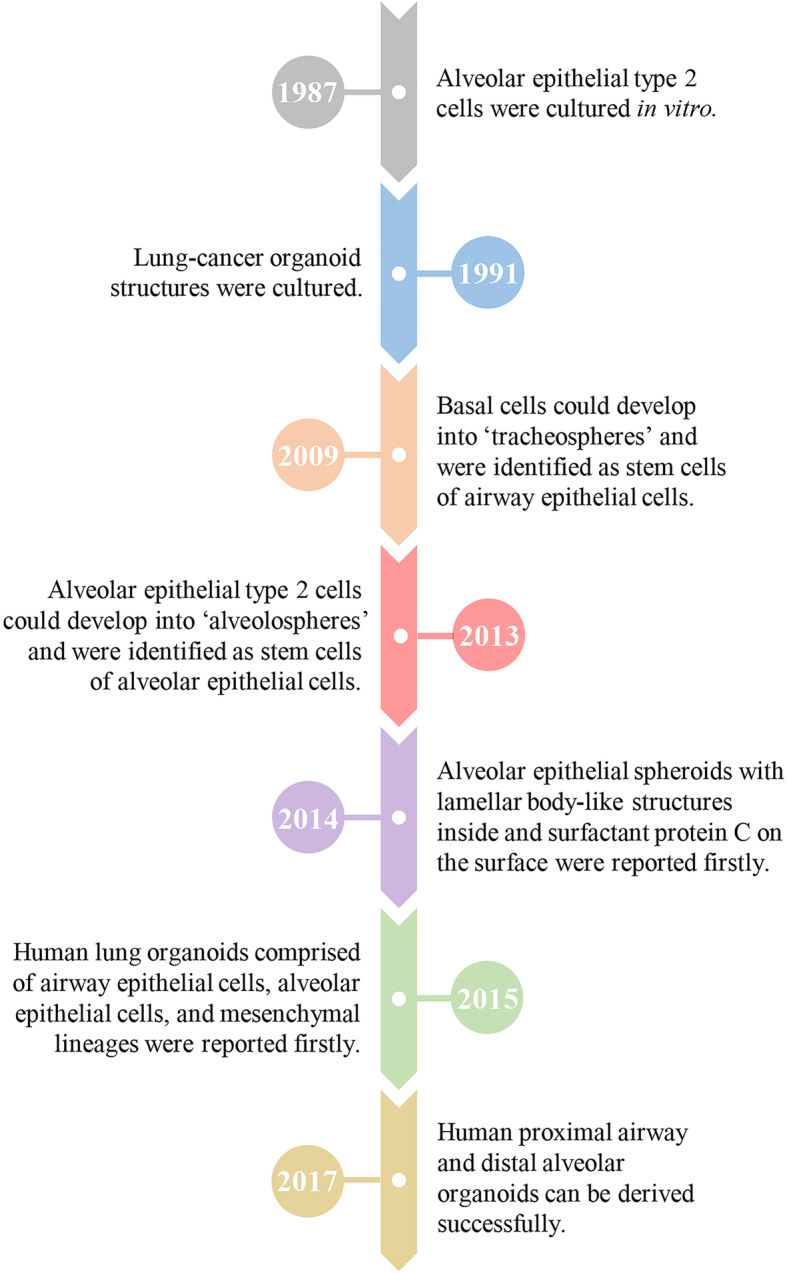
History of lung organoids. The important events in the history of the lung organoids are showed chronologically [11, 12, 14–19]

The history of lung organoids can be traced back to 1987. Jennings and colleagues cultured alveolar epithelial type 2 (AT2) cells in vitro, the daughter cells of which showed the morphological characteristics of the original cells [14]. In 1991, lung cancer cells were cultured in a simple system with a gas–medium interface, then reorganised and differentiated into organoid structures similar to the original tissues with regard to typical histological characteristics [15]. By 2009, mouse basal cells and human basal cells had been cultured in a clonal sphere-forming assay without stroma in vitro. They could self-renew and generate “tracheospheres” with the lumen, which included differentiated ciliated cells. Basal cells were identified as the stem cells of mice and humans, with two cell-surface markers: integrin alpha 6 and nerve growth factor receptor [16]. In 2013, Barkauskas and colleagues purified and seeded AT2 cells into a 3D co-culture system with primary platelet-derived growth factor receptor alpha^+^ (PDGFRα^+^) lung fibroblasts and readily derived self-renewing “alveolospheres”, which contained AT2 cells and alveolar epithelial type 1 (AT1) cells expressing a new surface marker: homeobox only protein X (HOPX). Simultaneously, they indicated that AT2 cells are stem cells in the adult lung [17]. In 2014, Jacob and coworkers identified and differentiated human alveolar epithelial progenitor cells (AEPs) with the surface marker carboxypeptidase M into alveolar epithelial spheroids, which showed lamellar body-like structures inside and expressed surfactant protein C (SFTPC) on the surface [18]. That method had never been reported previously. In 2015, human lung organoids arising from human pluripotent stem cells (hPSCs) were reported for the first time. Their structural features resembled those of the native lung and comprised cell types with proximal airway epithelium, distal alveolar epithelium, and mesenchymal lineages [19]. Since then, the technology of lung organoids has developed rapidly. To date, human proximal airway and distal alveolar organoids can be derived successfully [11, 12]. The important events in the history of the lung organoids are summarised chronologically in Fig. 1.

### Approaches of deriving lung organoids

Deriving lung organoids is an alterable and controllable process related to the starting cell types and culture microenvironments including culture media and culture systems. Organoids are clonal outgrowths of cells that are formed by the proliferation and differentiation of the original cells in vitro. They have similar biological characteristics and behaviours to their parent cells. Therefore, the primary factor in the production of lung organoids is the starting cell types, which can determine the final application of the lung organoids. Usually, the starting cells are pulmonary pluripotent stem/progenitor cells, adult stem cells, and embryonic stem cells. These starting cells can be identified and isolated through reporter lines and fluorescence-activated cell sorting and then plated on a culture system in vitro.

The other elements in the production of lung organoids are the culture microenvironments. Use of Matrigel in the cultivation environment is a major difference between organoid cultures and other cell cultures. Matrigel is an extract of a natural basement membrane that was first purified from Engelbreth–Holm–Swarm mouse sarcoma cells and used in early cultures of lung organoids [14, 20]. After decades of development, Matrigel is the most widely used product for generation of 3D lung organoids [11, 17, 19]. Matrigel contains a complex extracellular matrix (ECM) to provide cells with a support framework (just like in an organ) in vivo and promotes the growth and differentiation of cells.

Different research strategies have elicited different cultivation microenvironments for production of lung organoids. Barkauskas and colleagues established a 3D co-culture system with primary PDGFRα^+^ lung fibroblasts to produce more and rounder alveolospheres rapidly due to the trophic effect of fibroblasts [17]. Jacob and colleagues derived monolayered alveolospheres in 3D cultures without the support of mesenchyme [12]. The absence of stromal cells did not attenuate the competence of the iPSC-derived AT2 cells to proliferate and differentiate [12]. That culture system without supporting cells could reduce endogenous interference with the starting cells. de Carvalho and coworkers replaced Matrigel with collagen type 1 gels in the presence of other fibroblast growth factors (FGFs) and found the derived lung organoids expressed more markers of mature cells than those produced using Matrigel [21]. That finding provided additional options for the culture medium. To drive the stem cell to self-organise in vitro, signals which can act as development pathways in vivo are required that should be added to the 3D culture system, such as FGF7, FGF10, and bone morphogenetic protein (BMP)4 [13, 16, 17, 19].

Another culture system—the air–liquid interface (ALI) culture—can also support the proliferation and differentiation of airway stem cells. It is considered to be closely related to respiratory physiology, which preferentially recapitulates the pseudostratified mucociliary epithelial structure of airways [22], but restricts the spatial structure of trachea. To obtain lung organoids of realistic structure and function, these two systems—Matrigel and ALI—are combined as a “3D-ALI” system. It has been demonstrated that the function of multi-ciliated airway cells (MCACs) in lung organoids derived from hPSCs via the 3D-ALI system is superior to that of MCACs in a 3D culture [23]. If airway organoids must be generated, the 3D-ALI system can be given priority.

**Fig. 2:**
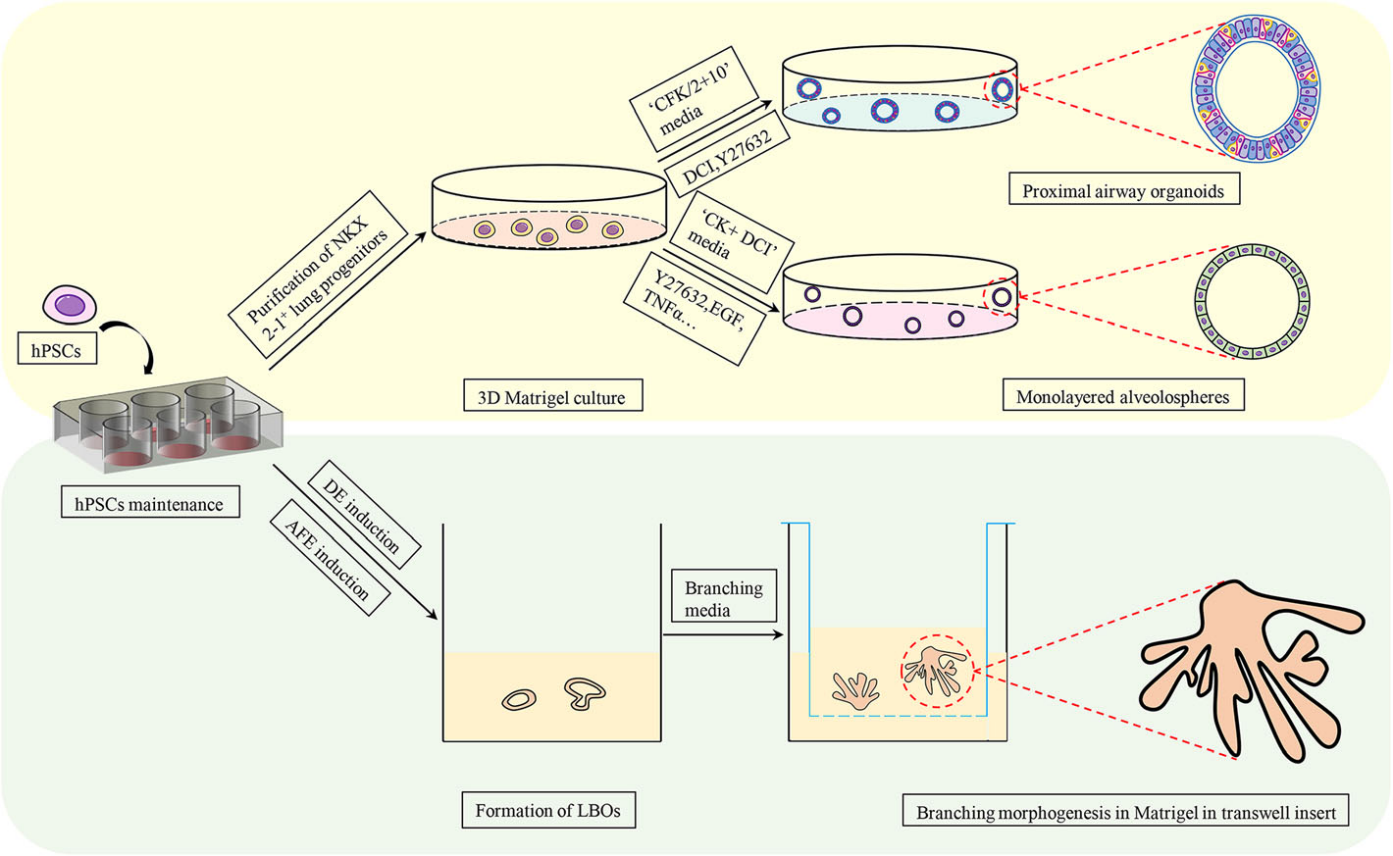
Generation of lung organoids from hPSCs. Different types of lung organoids can be harvested from directed differentiated hPSCs by controlling the compositions of the medium in a 3D Matrigel culture. For proximal airway organoids, “CFK media” can be chosen, which contains CHIR99021 (3 μM), recombinant human FGF (rhFGF) 10 (10 ng/mL), and recombinant human keratinocyte growth factor (rhKGF; 10 ng/mL), or another “2 + 10 media” containing rhFGF2 (250 ng/ml), rhFGF10 (100 ng/ml), DCI (50 nM dexamethasone, 0.1 mM 8-Bromoadenosine 3′,5′-cyclic monophosphate sodium salt and 0.1 mM 3-Isobutyl-1-methylxanthine), and Y-27632 [11]. For alveolar organoids, cells can be cultured in “CK + DCI” medium, which comprises CHIR99021 (3 μm) and rhKGF (10 ng/ml), additional Y-27632, and other growth factors or cytokines, such as EGF and TNFα [12]. Induced hPSCs, undergoing the steps of definitive endoderm (DE) and anterior foregut endoderm (AFE), can be treated with branching media to develop into lung bud organoids (LBOs). The latter could branch out and simulate lung tissues in Matrigel in 24-well transwell inserts if branching medium is added [13]

Hence, isolating and seeding starting cells into an appropriate culture system with the requisite signals, inducing them to undergo sequential developmental steps from the definitive endoderm to anterior foregut endoderm [24–26] and, finally, lung organoids, is the basic process of deriving lung organoids in vitro. McCauley and colleagues derived proximal airway organoids with the cell-surface markers NK2 homeobox 1^+^ (NKX2–1^+^) and tumour protein 63^+^ from hPSC-derived NKX2–1^+^ lung progenitors (Fig. 2). In addition, they used this strategy to establish airway organoids from cystic fibrosis patient-specific iPSCs. Those patient-specific airway organoids with a defect in forskolin-induced swelling could be adjusted by gene editing [11]. Moreover, Jacob and coworkers gave rise to functional alveolar organoids in a similar way by changing the starting cells and growth signals, and employed them to model alveolar disease [12] (Fig. 2). Furthermore, lung bud organoids (LBOs) containing the mesoderm and lung endoderm were generated from hPSCs. These LBOs could develop into branching airway and early alveolar structures in Matrigel and in vivo and recapitulated infection with the respiratory syncytial virus and fibrotic lung disease in vitro [13] (Fig. 2). These types of lung organoids had a near-physiological structure, retained the functions of the original tissue, and provided a powerful platform to model pulmonary diseases.

## Lung injury

### Epithelial repair after lung injury

The lung is composed of two main compartments: the airways (which include the proximal conducting airways and respiratory bronchioles) and distal alveoli. The lung undertakes the conduction and exchange of gas and communicates with the external environment continuously. The normal function of the lung is dependent upon the structural integrity of lung tissue. The adult lung is composed of multiple types of epithelial cells. The adult lung remains largely quiescent in homeostasis, and most of the cells that make up the epithelium display a relatively slow alternation of senescent cells [27, 28].

Following injury, the lung has a robust ability to repair and regenerate through distinct cell types. “Repair” is the process of self-renewing and restoring the damaged cells and tissues in an organ, including regeneration or fibroplasia [29] and, if both can occur, then the structure and function of the lung can recover [30]. “Regeneration” refers to generation of new cells and organisation into tissues from progenitor cells derived from de-differentiated tissue-resident cells or cells recruited from the circulation [27–29]. Various cell types (e.g. epithelial, mesenchymal) [17, 31–35] participate in regeneration of the lung epithelium which is controlled by Wnt [11], Notch [36], Hippo [37], and transforming growth factor (TGF) β signalling [38].

### Models of lung injury

Studying cell–cell interactions during repair after lung injury and the regulatory mechanism of the different signalling pathways is important. Models which can recapitulate processes in the lung from injury to recovery sensitively and accurately are required.

Mice are used commonly as model animals. The lung tissue of adult mice (or other model organisms) is removed through unilateral pneumonectomy to simulate acute lung injury and stimulate formation of the new alveoli that can compensate for the missing alveolar surface area [39–42]. Notably, in this model of lung regeneration, the whole lung and other body organs participate synergistically. This phenomenon is not conducive to exploring the details of damage repair because of several confounding factors.

Another common injury model used widely in experiments is treating mice with chemicals or biological agents (e.g. bleomycin) [17]. Bleomycin is a chemotherapeutic used to treat Hodgkin’s lymphoma, and its most feared side effect is pulmonary toxicity with fibrosis [43]. Over recent decades, bleomycin has been used widely to mimic lung injury in mice or rats [44]. Although the lung-fibrotic response can be observed ~ 14 days after administration of the initial dose (via intratracheal instillation) [45, 46], bleomycin is more appropriate to induce acute lung injury because fibrosis would resolve spontaneously beyond 28 days [45, 47]. To overcome this limitation, other dosing routes and regimens of bleomycin have been employed. For instance, the duration of lung damage can be prolonged to 24 weeks by a lower and repeated intratracheal-instillation dose of bleomycin, but the long time spent in modelling and dramatic mortality with this dosing regimen are major problems [48]. This model is contrary to the “3R” (reduce, refine, replace) theory of animal experiments.

Although mice provide promising opportunities for establishing a model of lung injury using the methods stated above due to their ease of handing, there remain many differences between human lungs and mice lungs. First, there is huge difference in the volume and weight of lungs. The human lung has thousand-fold more alveolar surface area and tidal volume than those of mice [49–51]. These remarkable differences present challenges for the distribution and exchange of gas and tissue repair. Second, the structure and cellular composition of the distal airways in mice are different from those in humans. In the latter, the intrapulmonary airways contain cartilaginous rings, bronchial blood support, submucosal glands, and pseudostratified epithelium. Within the pseudostratified epithelium of terminal and respiratory bronchioles, there are cytokeratins 5^+^ basal cells which constitute a population of pulmonary stem cells [52]. None of these cells are found in mouse lungs [30, 53]. In mice, there are no analogous counterparts to the respiratory bronchioles or bronchoalveolar duct junction (BADJ) which contain bronchoalveolar stem cells (BASCs) [54] that connect distal conducting airways to alveoli. But the BADJ has not been found in human lung and BASC-like cells have not been reported in human airways up to now. Hence, the origins of cells that take part in repair of the distal airways and alveoli may be not the same between humans and mice. Furthermore, some treatments that mice respond to may not work if applied to humans. For example, all-*trans* retinoic acid has been shown to promote the differentiation and regeneration of human alveolar epithelial stem cells in pre-clinical mouse models of COPD. However, clinical trials using retinoic acid or retinoids for emphysema patients have been unsuccessful [55].

Considering the critical differences between human lungs and mouse lungs, the latter may not be perfect models for researching human pulmonary diseases. Establishing more similar, sophisticated, and effective models which could narrow the gap from “laboratory bench to bedside” could be a better way to solve this problem. Lung organoids could be better choices.

## Lung organoids were used to study epithelial repair-associated cells after lung injury

### Lung organoids proved the presence of multiple dominate stem/progenitor cells during alveolar epithelial repair after injury

The alveolar epithelium is composed mainly of AT1 cells and AT2 cells. The former cover ~ 95% of the alveolar surface area (where gas exchange occurs). AT2 cells cover ~ 5% of the alveolar surface area and are mainly responsible for producing alveolar surfactants (which are irreplaceable in reducing the alveolar surface tension) [56]. In addition, AT2 cells are stem cells in adult lungs [17]. Choi and colleagues identified the damage-associated transient progenitors (DATPs) via lung organoid models. The DATPs are distinct AT2-lineage population and are required for AT2 cells differentiating to mature AT1 cells [57]. Further research indicated that AT2 cells are undergoing pre-alveolar type-1 transitional cell state (PATS) during the differentiation. However, persistence of the PATS was not conducive to alveolar epithelial repair [58]. In clonal 3D alveolar organoid assays, a subset of the human AT2 cells that is Wnt-responsive, of AEPs lineage, and which expresses the cell-surface marker transmembrane-4-L-six-family-1, can give rise to more and large organoids containing AT1 cells and AT2 cells [59, 60]. They act as primary functional AEP cells in the distal lung [59]. Recently, another team identified a population of adult distal lung epithelial progenitor cells with low Wnt/β-catenin activity. Their strong organoid-forming capacity means that they play an important role in the alveolar epithelial repair [61]. Moreover, 3D-ALI has been employed to study lineage-negative epithelial progenitors; these are another type of distal airway progenitor cells that differentiate into AT2 cells and rebuild the epithelium after epithelial damage [62]. Data on these alveolar organoids have shown that AT2 cells can self-renew and regenerate and that they aid alveolar epithelial repair following injury.

The contribution of AT1 cells to alveolar epithelial repair has not been studied in depth, but some studies have been meaningful. Both clonal 3D culture in vitro and lineage tracing following lung injury in mice revealed that Hopx^+^ AT1 cells proliferated and regenerated to AT2 cells during alveolar recovery [63]. Wang and colleagues investigated the clonal-formation capacity of Hopx^+^ AT1 cells in a 3D organoid culture system. They found the cellular plasticity of these Hopx^+^ and insulin-like growth factor-binding protein 2^−^ AT1 cells, which are subtypes of AT1 cells [64]. We postulate that Hopx^+^ AT1 cells have the potential of stem cells, they can renew and differentiate into AT2 cells during post injury alveolar regeneration, but the regulatory mechanism is poorly understood. Hence, lung organoids can be used for identifying the cell types and features [65] and increase insights into alveolar epithelial stem/progenitor cells during repair.

### Lung organoids confirmed that diverse airway epithelial stem/progenitor cells play important role in airway repair

The human trachea, bronchi, and bronchioles contain a pseudostratified ciliated columnar epithelium composed of multiple cell types (e.g. basal, ciliated, goblet, club). Basal cells constitute the primary stem cell population in the lung [52]. In mice, basal cells are present only in the trachea and proximal airways [53]. In vivo studies have shown that basal cells have self-renewal and pluripotent potentials and can differentiate into cells (e.g. ciliated, goblet, club) in the airway epithelium after injury [66]. Montoro and collaborators revealed that basal cells from mouse tracheas dissociated and differentiated in an ALI system and that subsequent treatment with recombinant murine interleukin (IL)-13 led to generation of goblet cells in the distal epithelium; this new subset of goblet cells may be related to airway epithelial injury [67]. Those data suggested a possible sub-line of basal cells which might participate in fluid regulation at the airway epithelial interface after injury [67].

Although basal cells play a major part in repair of the damaged airway epithelium, other epithelial cellular types can also aid repair as facultative stem/progenitor cells. Often, lung organoid technology is applied to investigate the functions of these cells. For instance, club cells that reside throughout the airway epithelium are facultative progenitor cells [68]. They can differentiate into an alveolar lineage induced by leucine-rich repeat-containing G protein-coupled receptor (Lgr) 5^+^ mesenchymal cells in a 3D co-culture organoid system [34]. Pulmonary neuroendocrine cells (PNECs) are neurosensory cells spread sparsely throughout the bronchial epithelium. PNECs can self-renew and differentiate into club cells and ciliated cells following lung injury [69, 70]. Lung organoid units from the lung tissues of mice and humans have been transplanted into immunodeficient mice and then grown into tissue-engineered lung containing multiple types of lung epithelial cells (including PNECs) [71]. The ALI culture has been used to demonstrate that myoepithelial cells from submucosal glands are “reserve” stem cells that can regenerate the airway epithelium in mice after severe injury to the airways [72]. Use of lung organoids has extended understanding of airway epithelial stem/progenitor cells during repair.

### Lung organoids found the contribution of distinct mesenchymal lineages to epithelial repair

Within the mammalian lung, the maintenance and regeneration of alveolar and airway epithelia are not isolated, and interact with the surrounding mesenchyme and ECM. The latter is the microenvironment that cells live and act in. The mesenchyme includes immune cells and fibroblasts. It plays a major part in interaction with the epithelium by providing complex signals that can regulate the behaviour of the epithelium in terms of homeostasis and following injury [73]. The injury–repair model in vivo shows that stromal components can survive damage [17], but the exact component that regulates the repair process is not known.

Macrophages are chiefly resident within the interstitium, and various cytokines (e.g. IL-13, IL-6, IL-1, tumour necrosis factor (TNF) α) are closely associated with the response of the epithelium after injury [74–76]. Hung and colleagues derived airway organoids from the mouse tracheal epithelial cells (MTECs) in ALI system, conducted the macrophage-epithelial repair assay, and demonstrated that macrophage intrinsic Trefoil factor 2/Wnt drove epithelial proliferation and barrier restoration [77]. Lechner and coworkers, using isolated macrophages from injured lung and bone-marrow derived macrophages from mice, co-cultured them with AT2 cells, respectively. They revealed that macrophages could support formation of organoids derived from AT2 cells and might promote the survival and proliferation of AT2 cells directly [33].

The integrated structure and function of AT2 cells and the adjacent pulmonary vasculature determine normal gas exchange [78]. Besides epithelial cells, the pulmonary endothelium also takes part in the repair process to recover normal gas exchange. The vascular-organoid assay demonstrated that the pulmonary vasculature includes endothelial progenitor cells which are vasculogenic and which can maintain functional endothelial microvascular specificity following lung damage [79].

The trophic effect of PDGFRα^+^ cells that support the epithelial development and alveolarisation in the lung has been shown [80]. Further research established a co-culture of organoids which confirmed that PDGFRα^+^ fibroblasts drive formation of larger and rounder alveolospheres from AT2 cells [17]. Another crucial aspect of distinct PDGFRα^+^ mesenchymal cells was demonstrated subsequently. Alveolar organoid assays were undertaken to define the Axin2-Pα^+^/PDGFRα^+^ lineage as a mesenchymal alveolar niche cell, which could promote the self-renewal and differentiation of AT2 cells [35]. Other studies using alveolar organoid assays discovered another lineage: Axin2^+^ myofibrogenic progenitor cells. The latter constitutively express Axin2 but little or no *Pdgfrα*, and preferentially facilitate myofibroblast production against injury [35]. Besides the crosstalk between Pdgfrα^+^ mesenchymal cells and the alveolar epithelium, other subtypes of Pdgfrα^+^ cell lineages act on the airway epithelium. Pdgfrα^+^ fibroblasts induced the differentiation of airway multi-ciliated cells from basal stem cells [75]. To identify the relationship between Lgr6^+^ mesenchymal cells and pulmonary epithelial cells, a 3D organoid co-culture system was generated. Results suggested that the subpopulations of mesenchymal cells were region-specific and one type (Lgr6^+^ cells) preferentially promoted differentiation and proliferation of the airways after lung injury [34]. The organoid assays described above suggest that PDGFRα^+^ mesenchymal lineages have complex effects upon epithelial repair after damage.

Epithelial repair after injury is a complex process of dynamic change involving cellular participation and regulation of signalling pathways. Using organoids to generate experimental models can recapitulate the physiological status in vivo while excluding the influence of other factors. Scholars can create diverse lung organoids (e.g. alveolar, airway) for various research purposes using 3D culture, multicellular co-culture systems, and 3D-ALI cultures. These organoids can be used to identify the cell types and their effects or cell–cell interactions during lung epithelium repair. Organoids can also deepen understanding of the regulatory mechanisms of epithelial repair following injury.

## Using lung organoids to explore the mechanisms of epithelial repair after injury

An intricate series of signalling pathways participate in epithelial regeneration following lung injury [11, 36–38]. Studies using lung organoids can illuminate the regulatory mechanisms of these signalling pathways in epithelial repair.

### Using lung organoids to study the mechanisms of alveolar epithelial repair

AT2 cells are a stem cell-containing population. They have a predominant role in repair after lung injury. Lung organoids can be employed to assess the relevant factors when AT2 cells self-renew and regenerate after injury. These factors manipulate different signalling pathways to affect the repair of the alveolar epithelium.

**Fig. 3:**
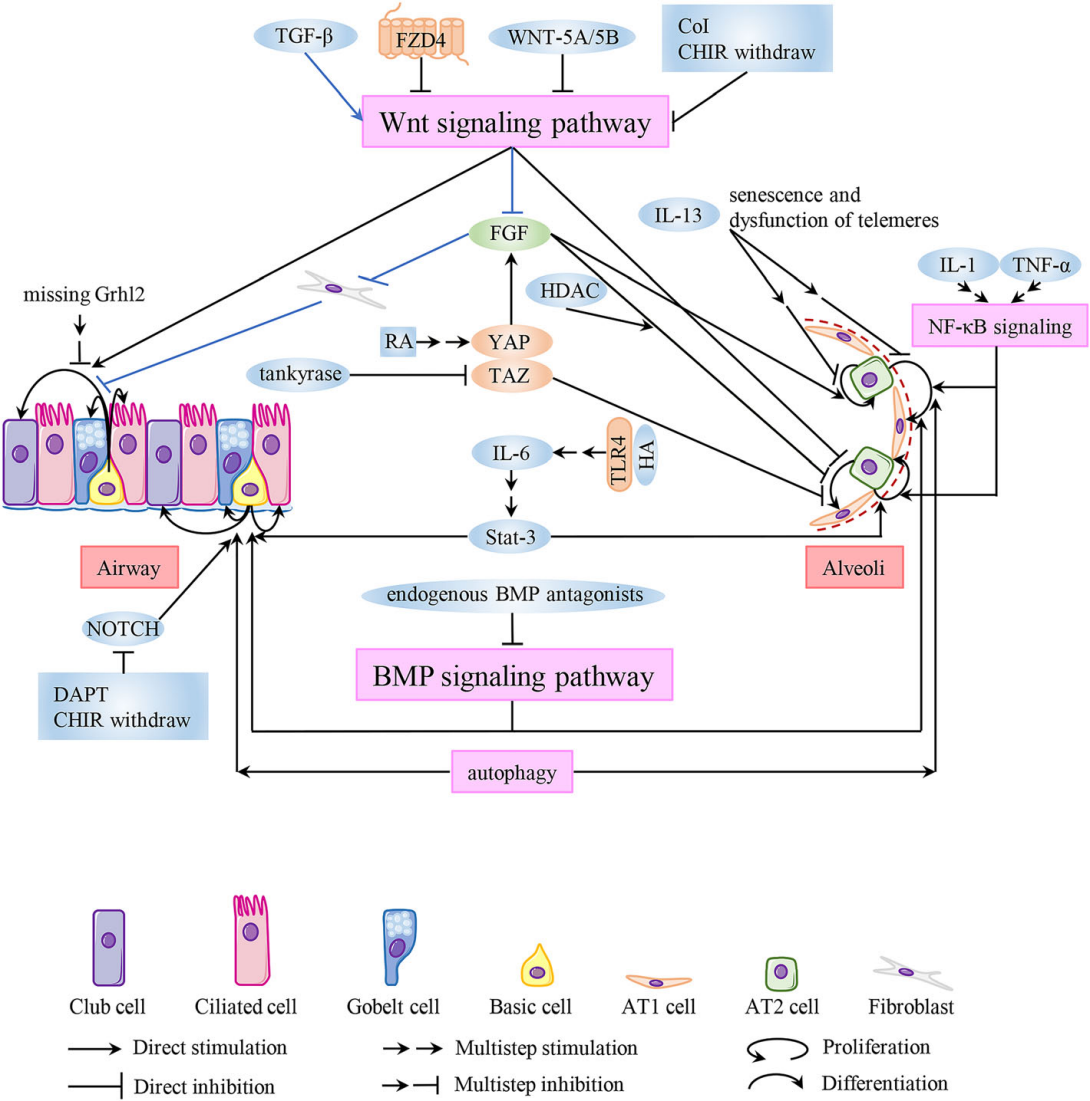
Mechanisms of airway and alveolar epithelial repair identified using lung organoids. The structure of the pseudostratified airway epidermis and alveolar epidermis in human lungs are shown. The main lineages and mechanisms involved in lung repair using lung organoids are revealed

Ng-Blichfeldt and colleagues found that proliferation of the distal lung epithelium (as reflected by the size and number of lung organoids) could be augmented by the retinoic acid pathway through activation of the yes-associated protein (YAP) pathway and epithelial–mesenchymal FGF signalling. However, epithelial differentiation was suppressed but could be rescued via inhibition of histone deacetylase [55] (Fig. 3). Sun and colleagues created a 3D alveolosphere organoid culture system with sorted AT2 cells and found that transcriptional coactivator with PDZ-binding motif (TAZ) was a critical factor affecting the differentiation of AT2 cells to AT1 cells, which could benefit the recovery of alveolar epithelial integrity following injury. The pro-differentiation effect of TAZ could be prevented by tankyrase inhibition [81] (Fig. 3).YAP/TAZ are the core elements in the Hippo pathway, and LaCanna and coworkers have demonstrated their effect on alveolar repair using other models [82].

Wnt signalling has critical roles in the differentiation and proliferation of lung epithelial stem/progenitor cells and helps to regulate proximodistal specification in the lung epithelium [83–86]. AT2 cells are the main AEPs which are Wnt-responsive, and maintenance of their stemness is also dependent on the Wnt signalling that is present after injury [59, 87]. Wnt ligands have specific transduction characteristics of the Wnt signalling pathway which affect the process of injury and repair in the lung epithelium. Lung organoids are the ideal models to explore the detailed mechanism of action. Wu and colleagues revealed that Wnt-5A and Wnt-5B inhibit generation of lung organoids. Wnt-5B preferentially represses the growth and differentiation of AEPs. They concluded that Wnt-5A/5B (which show high expression in COPD) lead to abnormal alveolar repair via negative regulation of the canonical Wnt signalling pathway in AEPs [88] (Fig. 3). Jacob and colleagues, using alveolar organoids, demonstrated that temporal regulation of Wnt activity could promote maturation of iPSC-derived AT2 cells [12]. It may be possible to use alveolar organoids to explore appropriate regulation of Wnt signalling in the AEPs of COPD patients and discover new treatment strategies.

Apart from Wnt ligands, another factor may inhibit repair of the alveolar epithelium through regulation of Wnt signalling. Skronska-Wasek and colleagues used a 3D organoid assay to elucidate that repression of expression of the Frizzled 4 receptor attenuated the competence of alveolar epithelial repair by Wnt/β-catenin [89] (Fig. 3). Recently, ex vivo organoid platforms combined with in vivo models of lung repair and other methods have been used to demonstrate that IL-13 damaged the self-renewal and differentiation ability of AT2 cells in mice and humans [90] (Fig. 3). Use of alveolar organoids also showed that other cytokines—IL-1 and TNFα—act upon the nuclear factor-kappa B pathway to enhance proliferation of AT2 cells and maintain their differentiation, which have essential roles in alveolar regeneration [76] (Fig. 3). In addition, the capacity of AT2 cells with short telomeres from mice to support formation of alveolar organoid colonies is limited; it is closely related to senescence and the immune response induced by dysfunction of telomeres in AT2 cells which upregulate alveolar epithelial defects [91] (Fig. 3).

### Using lung organoids to explore the mechanisms of airway epithelial repair

Lung epithelial progenitor cells regenerate and differentiate into ciliated cells and other cell types to counteract the loss of the airway epithelium after injury. Organoids are useful models for investigating the regulatory mechanisms during airway epithelial repair derived from lung epithelial progenitor cells.

Wnt signalling also affects regeneration of the airway epithelium. McCauly and collaborators derived airway organoids from human iPSCs. They identified that the fate of lung tissue tended to develop proximally following a decrease in Wnt activity. Wnt signalling has an intrinsic effect on NKX2–1^+^ lung epithelial progenitor cells [11]. de Cavalho and colleagues demonstrated, from multiple perspectives, that changing the culture conditions (replacing Matrigel in the 3D culture system with collagen-I and retreatment with CHIR99021 (CHIR) (Wnt agonist)) was conducive to inducing the differentiation and maturation of multiple lineages, including club cells and ciliated cells [21] (Fig. 3). At the same time, WNT signal would also indirectly affect the repair of injured airway epithelium. TGF-β has been shown to disrupt the ability of fibroblasts to sustain organoid formation derived from the lung epithelium, which hampers epithelium regeneration after injury [92]. TGF-β alters expression of the downstream target genes of Wnt/β-catenin and diminishes FGF expression (which is required for fibroblast maintenance) [92] (Fig. 3). Taking the aforementioned data on Wnt signalling in lung organoids together shows that use of lung organoids as an in vitro model system can manipulate Wnt signalling to regulate differentiation of the proximodistal specification of lung development. This strategy could facilitate establishment of appropriate organoid models for pulmonary injury based on research requirements (e.g. airway disease, alveolar disease). These different types of lung organoids could be applied to mechanistic research of lung disease and drug screening, and promote “precision medicine” for lung diseases.

In addition to Wnt signalling, Notch signalling also participates in the proximodistal specification of lung development. Using genome-editing methods in ALI and 3D organoid cultures, the grainyhead-like transcription factor 2 (*Grhl2*) was identified as a coordinator having various roles via multiple downstream effectors [93]. Organoid morphogenesis and differentiation of basal cells into ciliated cells are prevented by the loss of *Grhl2* expression, whereas expression of Notch and the genes involved in ciliogenesis (e.g. Multicilin, regulatory factor X 2, and myeloblastosis oncogene) is downregulated [93] (Fig. 3). If CHIR is removed, suppression of the Notch signal with the inhibitor N-[N-(3,5-difluorophenacetyl)-L-alanyl]-S-phenylglycine t-butyl ester could induce the lung epithelium to form proximal lung organiods [21] (Fig. 3).

Use of organoids in research has shown that some pathways regulate alveolar and airway epithelial repair in a similar way. Multiple studies using lung organoids demonstrated that the IL-6/signal transducer and activator of transcription 3 (Stat3) pathway upregulated differentiation of basal cells into ciliated cells and secretory cells [75]. Similarly, the IL-6/Stat3 pathway could promote self-renewal of AT2 cells but requires interactions between Toll-like receptor and the glycosaminoglycan hyaluronan in the ECM [7, 35]. The IL-6/Stat3 pathway benefits recovery of the lung epithelium. In addition, the function of the BMP signalling pathway and its antagonists was reported by two research teams using organoids derived from basal cells or AT2 cells, respectively. Taking their findings together, it was elucidated that the effect of the BMP signalling pathway was dependent upon lung status. At steady state, the BMP signalling reduces the trophic support of PDGFRα^+^ cells and restricts proliferation of epithelial in the lung. The BMP may have a negative influence on epithelial repair. This function fails early in repair due to upregulation of expression of endogenous BMP antagonists following injury, and then the “packing” of epithelial cells increases [94, 95] (Fig. 3). Besides these signalling pathways, autophagy has an essential role in satisfying the energy needs and promoting lung epithelial regeneration after injury by reprogramming the metabolism of epithelial progenitor cells [96, 97] (Fig. 3).

In conclusion, lung organoids are excellent models for researching epithelial repair after damage. Their use results in identification of the subtypes of epithelial stem/progenitor cells, and the factors and mechanisms regulating signalling pathways. Such data provide solid evidence for further study of epithelial repair after lung injury.

## Conclusions and future directions

In the past two decades, lung organoid technology has developed rapidly. Lung organoids are useful tools to expand understanding of lung injury by identifying the epithelial stem/progenitor cells associated with epithelial recovery and demonstrating the related factors which can manipulate the signalling pathways to improve or inhibit epithelial regeneration. Such information will allow deeper insight into the pathological processes and drug targets of pulmonary diseases.

However, many limitations exist. First, the training technology must be objective and standardised. Originating from the same cell, different organoids can be obtained by changing the culture conditions [11, 12]. This strategy allows derivation of organoids depending on the research purpose but, simultaneously, may also affect the repeatability of the organoids, resulting in biased research results. Exploitation of a stable ECM may be the solution to this problem. The branching airway is a critical structure to achieve air conduction in the lung. Lung organoids are often spherical and do not completely simulate this morphological structure. This problem may require enhanced nutritional support or optimised media compositions for organoids in culture systems and extended culture times. Bioengineering technology, such as 3D bioprinting technology [98], could be combined with organoid morphogenesis to produce structurally complete organoids in a short time.

Furthermore, the crosstalk between multiple signalling pathways in epithelial repair needs further confirmation. This is a complex relationship involving multiple cell types besides the epithelium. Currently, the relationship between these cell types has not been completely elucidated by using lung organoids. Addition of immune cells or vascular endothelial cells in an orderly manner while deriving lung organoids, and culturing them together to reproduce the microenvironment of epithelial repair in vitro, could be applied to better observe the relationship between cells and cells, or cells and the ECM, during epithelial regeneration. Organoid technology of other organs is also developing rapidly [99–104].

SARS-CoV-2 infection is spreading worldwide, and the organoids show an important role in the study of the new virus. The researchers combined lung organoids and other organoids together to mimic the situation post infection of SARS-CoV-2 in the patients similarly [105]. Lung organoids and other organoids, what is more, the multi-tissue organ-on-a-chip platform (including multiple human-derived organoids), could be used for revealing the pathological process of the organs after infection of SARS-CoV-2, screening the candidate drugs, and developing and evaluating vaccines for safety and efficacy [106–112]. In conclusion, a combination of lung organoids and various technologies could enable more precise understanding of lung epithelial repair and promote treatment of pulmonary diseases.

## Data Availability

All data supporting this article are included within the article.

## Abbreviations

SARS-CoV: Severe acute respiratory syndrome coronavirus
COPD: Chronic obstructive pulmonary disease
IPF: Idiopathic pulmonary fibrosis
3D: Three-dimensional
AT2: Alveolar epithelial type 2
PDGFRα^+^: Platelet-derived growth factor receptor alpha+
AT1: Alveolar epithelial type 1
HOPX: Homeobox only protein X
AEPs: Alveolar epithelial progenitor cells
SFTPC: Surfactant protein C
hPSCs: Human pluripotent stem cells
ECM: Extracellular matrix
FGFs: Fibroblast growth factors
BMP: Bone morphogenetic protein
ALI: Air–liquid interface
MCACs: Multi-ciliated airway cells
NKX2–1^+^: NK2 homeobox 1+
LBOs: Lung bud organoids
TGF: Transforming growth factor
BADJ: Bronchioles or bronchoalveolar duct junction
BASCs: Bronchoalveolar stem cells
DATPs: Damage-associated transient progenitors
PATS: Pre-alveolar type-1 transitional cell state
IL: Interleukin
Lgr: Leucine-rich repeat-containing G protein-coupled receptor
PNECs: Pulmonary neuroendocrine cells
TNF: Tumour necrosis factor
MTECs: Mouse tracheal epithelial cells
YAP: Yes-associated protein
TAZ: Transcriptional coactivator with PDZ-binding motif
CHIR: CHIR99021
Grhl2: Grainyhead-like transcription factor 2
Stat3: Signal transducer and activator of transcription 3

## References

[1] Tan KS, Andiappan AK, Lee B, Yan Y, Liu J, Tang SA (2019). RNA sequencing of H3N2 influenza virus-infected human nasal epithelial cells from multiple subjects reveals molecular pathways associated with tissue injury and complications. Cells..

[2] Zhu N, Zhang D, Wang W, Li X, Yang B, Song J (2020). A novel coronavirus from patients with pneumonia in China, 2019. N Engl J Med.

[3] Zuo WL, Yang J, Gomi K, Chao I, Crystal RG, Shaykhiev R (2017). EGF-amphiregulin interplay in airway stem/progenitor cells links the pathogenesis of smoking-induced lesions in the human airway epithelium. Stem Cells.

[4] Ghosh M, Miller YE, Nakachi I, Kwon JB, Baron AE, Brantley AE (2018). Exhaustion of airway basal progenitor cells in early and established chronic obstructive pulmonary disease. Am J Respir Crit Care Med.

[5] Zhao H, Mu X, Zhang X, You Q (2020). Lung cancer inhibition by betulinic acid nanoparticles via adenosine 5′-triphosphate (ATP)-binding cassette transporter G1 gene downregulation. Med Sci Monit.

[6] Bonser LR, Erle DJ (2019). The airway epithelium in asthma. Adv Immunol.

[7] Liang J, Zhang Y, Xie T, Liu N, Chen H, Geng Y (2016). Hyaluronan and TLR4 promote surfactant-protein-C-positive alveolar progenitor cell renewal and prevent severe pulmonary fibrosis in mice. Nat Med.

[8] Israel-Biet D, Juvin K, Dang Tran K, Badia A, Cazes A, Delclaux C (2014). Idiopathic pulmonary fibrosis: diagnosis and treatment in 2013. Rev Pneumol Clin.

[9] Clevers H (2016). Modeling development and disease with organoids. Cell..

[10] Lancaster MA, Knoblich JA (2014). Organogenesis in a dish: modeling development and disease using organoid technologies. Science..

[11] McCauley KB, Hawkins F, Serra M, Thomas DC, Jacob A, Kotton DN (2017). Efficient derivation of functional human airway epithelium from pluripotent stem cells via temporal regulation of Wnt signaling. Cell Stem Cell.

[12] Jacob A, Morley M, Hawkins F, McCauley KB, Jean JC, Heins H (2017). Differentiation of human pluripotent stem cells into functional lung alveolar epithelial cells. Cell Stem Cell.

[13] Chen Y-W, Huang SX, de Carvalho ALRT, Ho S-H, Islam MN, Volpi S (2017). A three-dimensional model of human lung development and disease from pluripotent stem cells. Nat Cell Biol.

[14] Shannon JM, Mason RJ, Jennings SD (1987). Functional differentiation of alveolar type ii epithelial cells in vitro: effects of cell shape, cell-matrix interactions and cell-cell interactions. Biochim Biophys Acta.

[15] Kopf-Maier P, Zimmermann B (1991). Organoid reorganization of human tumors under in vitro conditions. Cell Tissue Res.

[16] Rock JR, Onaitis MW, Rawlins EL, Lu Y, Clark CP, Xue Y (2009). Basal cells as stem cells of the mouse trachea and human airway epithelium. Proc Natl Acad Sci U S A.

[17] Barkauskas CE, Cronce MJ, Rackley CR, Bowie EJ, Keene DR, Stripp BR (2013). Type 2 alveolar cells are stem cells in adult lung. J Clin Invest.

[18] Gotoh S, Ito I, Nagasaki T, Yamamoto Y, Konishi S, Korogi Y (2014). Generation of alveolar epithelial spheroids via isolated progenitor cells from human pluripotent stem cells. Stem Cell Rep..

[19] Dye BR, Hill DR, Ferguson MA, Tsai YH, Nagy MS, Dyal R (2015). In vitro generation of human pluripotent stem cell derived lung organoids. Elife..

[20] Orkin RW, Gehron P, McGoodwin EB, Martin GR, Valentine T, Swarm R (1977). A murine tumor producing a matrix of basement membrane. J Exp Med.

[21] de Carvalho A, Strikoudis A, Liu HY, Chen YW, Dantas TJ, Vallee RB (2019). Glycogen synthase kinase 3 induces multilineage maturation of human pluripotent stem cell-derived lung progenitors in 3D culture. Development.

[22] Fulcher ML, Randell SH (2013). Human nasal and tracheo-bronchial respiratory epithelial cell culture. Meth Mol Biol.

[23] Konishi S, Gotoh S, Tateishi K, Yamamoto Y, Korogi Y, Nagasaki T (2016). Directed induction of functional multi-ciliated cells in proximal airway epithelial spheroids from human pluripotent stem cells. Stem Cell Rep..

[24] Huang SX, Islam MN, O'Neill J, Hu Z, Yang YG, Chen YW (2014). Efficient generation of lung and airway epithelial cells from human pluripotent stem cells. Nat Biotechnol.

[25] Green MD, Chen A, Nostro MC, d'Souza SL, Schaniel C, Lemischka IR (2011). Generation of anterior foregut endoderm from human embryonic and induced pluripotent stem cells. Nat Biotechnol.

[26] Huang SX, Green MD, de Carvalho AT, Mumau M, Chen YW, D'Souza SL (2015). The in vitro generation of lung and airway progenitor cells from human pluripotent stem cells. Nat Protoc.

[27] Whitsett JA, Kalin TV, Xu Y, Kalinichenko VV (2019). Building and regenerating the lung cell by cell. Physiol Rev.

[28] Hogan BL, Barkauskas CE, Chapman HA, Epstein JA, Jain R, Hsia CC (2014). Repair and regeneration of the respiratory system: complexity, plasticity, and mechanisms of lung stem cell function. Cell Stem Cell.

[29] Bolte C, Kalin TV, Kalinichenko VV (2020). Molecular, cellular, and bioengineering approaches to stimulate lung regeneration after injury. Semin Cell Dev Biol.

[30] Basil MC, Katzen J, Engler AE, Guo M, Herriges MJ, Kathiriya JJ (2020). The cellular and physiological basis for lung repair and regeneration: past, present, and future. Cell Stem Cell.

[31] Rafii S, Cao Z, Lis R, Siempos II, Chavez D, Shido K (2015). Platelet-derived SDF-1 primes the pulmonary capillary vascular niche to drive lung alveolar regeneration. Nat Cell Biol.

[32] Cao Z, Ye T, Sun Y, Ji G, Shido K, Chen Y (2017). Targeting the vascular and perivascular niches as a regenerative therapy for lung and liver fibrosis. Sci Transl Med.

[33] Lechner AJ, Driver IH, Lee J, Conroy CM, Nagle A, Locksley RM (2017). Recruited monocytes and type 2 immunity promote lung regeneration following pneumonectomy. Cell Stem Cell.

[34] Lee JH, Tammela T, Hofree M, Choi J, Marjanovic ND, Han S (2017). Anatomically and functionally distinct lung mesenchymal populations marked by Lgr5 and Lgr6. Cell..

[35] Zepp JA, Zacharias WJ, Frank DB, Cavanaugh CA, Zhou S, Morley MP (2017). Distinct mesenchymal lineages and niches promote epithelial self-renewal and myofibrogenesis in the lung. Cell..

[36] Mori M, Mahoney JE, Stupnikov MR, Paez-Cortez JR, Szymaniak AD, Varelas X (2015). Notch3-jagged signaling controls the pool of undifferentiated airway progenitors. Development..

[37] Gokey JJ, Sridharan A, Xu Y, Green J, Carraro G, Stripp BR (2018). Active epithelial Hippo signaling in idiopathic pulmonary fibrosis. JCI Insight.

[38] Aschner Y, Downey GP (2016). Transforming growth factor-beta: master regulator of the respiratory system in health and disease. Am J Respir Cell Mol Biol.

[39] Buhain WJ, Brody JS (1973). Compensatory growth of the lung following pneumonectomy. J Appl Physiol.

[40] Dane DM, Yilmaz C, Estrera AS, Hsia CC (2013). Separating in vivo mechanical stimuli for postpneumonectomy compensation: Physiological assessment. J Appl Physiol (1985).

[41] Ravikumar P, Yilmaz C, Bellotto DJ, Dane DM, Estrera AS, Hsia CC (2013). Separating in vivo mechanical stimuli for postpneumonectomy compensation: Imaging and ultrastructural assessment. J Appl Physiol (1985).

[42] Ravikumar P, Yilmaz C, Dane DM, Bellotto DJ, Estrera AS, Hsia CC (2014). Defining a stimuli-response relationship in compensatory lung growth following major resection. J Appl Physiol (1985).

[43] Udupa CBK, Koteshwar P, Udupa KS (2019). Bleomycin in Hodgkin's lymphoma - a boon or a bane? - a retrospective study of bleomycin pulmonary toxicity in Hodgkin's lymphoma. Indian J Palliat Care.

[44] Carrington R, Jordan S, Pitchford SC, Page CP (2018). Use of animal models in ipf research. Pulm Pharmacol Ther.

[45] Moeller A, Ask K, Warburton D, Gauldie J, Kolb M (2008). The bleomycin animal model: a useful tool to investigate treatment options for idiopathic pulmonary fibrosis?. Int J Biochem Cell Biol.

[46] Chaudhary NI, Schnapp A, Park JE (2006). Pharmacologic differentiation of inflammation and fibrosis in the rat bleomycin model. Am J Respir Crit Care Med.

[47] Moore BB, Hogaboam CM (2008). Murine models of pulmonary fibrosis. Am J Physiol Lung Cell Mol Physiol..

[48] Peng R, Sridhar S, Tyagi G, Phillips JE, Garrido R, Harris P (2013). Bleomycin induces molecular changes directly relevant to idiopathic pulmonary fibrosis: a model for “active” disease. Plos One.

[49] Knust J, Ochs M, Gundersen HJ, Nyengaard JR (2009). Stereological estimates of alveolar number and size and capillary length and surface area in mice lungs. Anat Rec (Hoboken).

[50] Irvin CG, Bates JH (2003). Measuring the lung function in the mouse: the challenge of size. Respir Res.

[51] Persson CG (2002). Con: mice are not a good model of human airway disease. Am J Respir Crit Care Med.

[52] Nadkarni RR, Abed S, Draper JS (2018). Stem cells in pulmonary disease and regeneration. Chest..

[53] Heng WS, Gosens R, Kruyt FAE (2019). Lung cancer stem cells: origin, features, maintenance mechanisms and therapeutic targeting. Biochem Pharmacol.

[54] Kim CF, Jackson EL, Woolfenden AE, Lawrence S, Babar I, Vogel S (2005). Identification of bronchioalveolar stem cells in normal lung and lung cancer. Cell..

[55] Ng-Blichfeldt JP, Schrik A, Kortekaas RK, Noordhoek JA, Heijink IH, Hiemstra PS (2018). Retinoic acid signaling balances adult distal lung epithelial progenitor cell growth and differentiation. EBioMedicine..

[56] Chen F, Fine A (2016). Stem cells in lung injury and repair. Am J Pathol.

[57] Choi J, Park JE, Tsagkogeorga G, Yanagita M, Koo BK, Han N (2020). Inflammatory signals induce at2 cell-derived damage-associated transient progenitors that mediate alveolar regeneration. Cell Stem Cell.

[58] Kobayashi Y, Tata A, Konkimalla A, Katsura H, Lee RF, Ou J (2020). Persistence of a regeneration-associated, transitional alveolar epithelial cell state in pulmonary fibrosis. Nat Cell Biol.

[59] Zacharias WJ, Frank DB, Zepp JA, Morley MP, Alkhaleel FA, Kong J (2018). Regeneration of the lung alveolus by an evolutionarily conserved epithelial progenitor. Nature..

[60] Barkauskas CE, Chung MI, Fioret B, Gao X, Katsura H, Hogan BL (2017). Lung organoids: current uses and future promise. Development..

[61] Hu Y, Ng-Blichfeldt JP, Ota C, Ciminieri C, Ren W, Hiemstra PS (2020). Wnt/beta-catenin signaling is critical for regenerative potential of distal lung epithelial progenitor cells in homeostasis and emphysema. Stem Cells.

[62] Xi Y, Kim T, Brumwell AN, Driver IH, Wei Y, Tan V (2017). Local lung hypoxia determines epithelial fate decisions during alveolar regeneration. Nat Cell Biol.

[63] Jain R, Barkauskas CE, Takeda N, Bowie EJ, Aghajanian H, Wang Q (2015). Plasticity of Hopx(+) type i alveolar cells to regenerate type ii cells in the lung. Nat Commun.

[64] Wang Y, Tang Z, Huang H, Li J, Wang Z, Yu Y (2018). Pulmonary alveolar type i cell population consists of two distinct subtypes that differ in cell fate. Proc Natl Acad Sci U S A.

[65] Evans KV, Lee JH (2020). Alveolar wars: the rise of in vitro models to understand human lung alveolar maintenance, regeneration, and disease. Stem Cells Transl Med.

[66] Kotton DN, Morrisey EE (2014). Lung regeneration: mechanisms, applications and emerging stem cell populations. Nat Med.

[67] Montoro DT, Haber AL, Biton M, Vinarsky V, Lin B, Birket SE (2018). A revised airway epithelial hierarchy includes CFTR-expressing ionocytes. Nature..

[68] Snyder JC, Reynolds SD, Hollingsworth JW, Li Z, Kaminski N, Stripp BR (2010). Clara cells attenuate the inflammatory response through regulation of macrophage behavior. Am J Respir Cell Mol Biol.

[69] Garg A, Sui P, Verheyden JM, Young LR, Sun X (2019). Consider the lung as a sensory organ: a tip from pulmonary neuroendocrine cells. Curr Top Dev Biol.

[70] Ouadah Y, Rojas ER, Riordan DP, Capostagno S, Kuo CS, Krasnow MA (2019). Rare pulmonary neuroendocrine cells are stem cells regulated by Rb, p53, and notch. Cell..

[71] Trecartin A, Danopoulos S, Spurrier R, Knaneh-Monem H, Hiatt M, Driscoll B (2016). Establishing proximal and distal regional identities in murine and human tissue-engineered lung and trachea. Tissue Eng Part C Meth.

[72] Lynch TJ, Anderson PJ, Rotti PG, Tyler SR, Crooke AK, Choi SH (2018). Submucosal gland myoepithelial cells are reserve stem cells that can regenerate mouse tracheal epithelium. Cell Stem Cell.

[73] Lloyd CM, Marsland BJ (2017). Lung homeostasis: influence of age, microbes, and the immune system. Immunity..

[74] Kuperman DA, Huang X, Koth LL, Chang GH, Dolganov GM, Zhu Z (2002). Direct effects of interleukin-13 on epithelial cells cause airway hyperreactivity and mucus overproduction in asthma. Nat Med.

[75] Tadokoro T, Wang Y, Barak LS, Bai Y, Randell SH, Hogan BL (2014). IL-6/STAT3 promotes regeneration of airway ciliated cells from basal stem cells. Proc Natl Acad Sci U S A.

[76] Katsura H, Kobayashi Y, Tata PR, Hogan BLM (2019). IL-1 and TNFalpha contribute to the inflammatory niche to enhance alveolar regeneration. Stem Cell Rep.

[77] Hung LY, Sen D, Oniskey TK, Katzen J, Cohen NA, Vaughan AE (2019). Macrophages promote epithelial proliferation following infectious and non-infectious lung injury through a trefoil factor 2-dependent mechanism. Mucosal Immunol.

[78] Morrisey EE, Hogan BL (2010). Preparing for the first breath: genetic and cellular mechanisms in lung development. Dev Cell.

[79] Alvarez DF, Huang L, King JA, ElZarrad MK, Yoder MC, Stevens T (2008). Lung microvascular endothelium is enriched with progenitor cells that exhibit vasculogenic capacity. Am J Physiol Lung Cell Mol Physiol.

[80] Bostrom H, Gritli-Linde A, Betsholtz C (2002). PDGF-a/PDGF alpha-receptor signaling is required for lung growth and the formation of alveoli but not for early lung branching morphogenesis. Dev Dyn.

[81] Sun T, Huang Z, Zhang H, Posner C, Jia G, Ramalingam TR (2019). Taz is required for lung alveolar epithelial cell differentiation after injury. JCI Insight..

[82] LaCanna R, Liccardo D, Zhang P, Tragesser L, Wang Y, Cao T (2019). Yap/Taz regulate alveolar regeneration and resolution of lung inflammation. J Clin Invest.

[83] Malta TM, Sokolov A, Gentles AJ, Burzykowski T, Poisson L, Weinstein JN (2018). Machine learning identifies stemness features associated with oncogenic dedifferentiation. Cell..

[84] Tammela T, Sanchez-Rivera FJ, Cetinbas NM, Wu K, Joshi NS, Helenius K (2017). A Wnt-producing niche drives proliferative potential and progression in lung adenocarcinoma. Nature..

[85] Hashimoto S, Chen H, Que J, Brockway BL, Drake JA, Snyder JC (2012). Beta-catenin-sox2 signaling regulates the fate of developing airway epithelium. J Cell Sci.

[86] Volckaert T, Campbell A, Dill E, Li C, Minoo P, De Langhe S (2013). Localized Fgf10 expression is not required for lung branching morphogenesis but prevents differentiation of epithelial progenitors. Development..

[87] Nabhan AN, Brownfield DG, Harbury PB, Krasnow MA, Desai TJ (2018). Single-cell Wnt signaling niches maintain stemness of alveolar type 2 cells. Science..

[88] Wu X, van Dijk EM, Ng-Blichfeldt JP, Bos IST, Ciminieri C, Konigshoff M (2019). Mesenchymal WNT-5A/5B signaling represses lung alveolar epithelial progenitors. Cells..

[89] Skronska-Wasek W, Mutze K, Baarsma HA, Bracke KR, Alsafadi HN, Lehmann M (2017). Reduced frizzled receptor 4 expression prevents Wnt/beta-catenin-driven alveolar lung repair in chronic obstructive pulmonary disease. Am J Respir Crit Care Med.

[90] Glisinski KM, Schlobohm AJ, Paramore SV, Birukova A, Moseley MA, Foster MW (2020). Interleukin-13 disrupts type 2 pneumocyte stem cell activity. JCI Insight..

[91] Alder JK, Barkauskas CE, Limjunyawong N, Stanley SE, Kembou F, Tuder RM (2015). Telomere dysfunction causes alveolar stem cell failure. Proc Natl Acad Sci U S A.

[92] Ng-Blichfeldt JP, de Jong T, Kortekaas RK, Wu X, Lindner M, Guryev V (2019). TGF-beta activation impairs fibroblast ability to support adult lung epithelial progenitor cell organoid formation. Am J Physiol Lung Cell Mol Physiol..

[93] Gao X, Bali AS, Randell SH, Hogan BL (2015). GRHL2 coordinates regeneration of a polarized mucociliary epithelium from basal stem cells. J Cell Biol.

[94] Tadokoro T, Gao X, Hong CC, Hotten D, Hogan BL (2016). BMP signaling and cellular dynamics during regeneration of airway epithelium from basal progenitors. Development..

[95] Chung MI, Bujnis M, Barkauskas CE, Kobayashi Y, Hogan BLM (2018). Niche-mediated BMP/SMAD signaling regulates lung alveolar stem cell proliferation and differentiation. Development.

[96] Li K, Li M, Li W, Yu H, Sun X, Zhang Q (2019). Airway epithelial regeneration requires autophagy and glucose metabolism. Cell Death Dis.

[97] Li X, Wu J, Sun X, Wu Q, Li Y, Li K (2020). Autophagy reprograms alveolar progenitor cell metabolism in response to lung injury. Stem Cell Rep..

[98] Estermann M, Bisig C, Septiadi D, Petri-Fink A, Rothen-Rutishauser B (2020). Bioprinting for human respiratory and gastrointestinal in vitro models. Meth Mol Biol..

[99] Amin ND, Pasca SP (2018). Building models of brain disorders with three-dimensional organoids. Neuron..

[100] Mills RJ, Parker BL, Quaife-Ryan GA, Voges HK, Needham EJ, Bornot A (2019). Drug screening in human PSC-cardiac organoids identifies pro-proliferative compounds acting via the mevalonate pathway. Cell Stem Cell.

[101] Takebe T, Sekine K, Kimura M, Yoshizawa E, Ayano S, Koido M (2017). Massive and reproducible production of liver buds entirely from human pluripotent stem cells. Cell Rep.

[102] Yan HHN, Siu HC, Law S, Ho SL, Yue SSK, Tsui WY (2018). A comprehensive human gastric cancer organoid biobank captures tumor subtype heterogeneity and enables therapeutic screening. Cell Stem Cell.

[103] Kraiczy J, Ross ADB, Forbester JL, Dougan G, Vallier L, Zilbauer M (2019). Genome-wide epigenetic and transcriptomic characterization of human-induced pluripotent stem cell-derived intestinal epithelial organoids. Cell Mol Gastroenterol Hepatol.

[104] Morizane R, Bonventre JV (2017). Kidney organoids: a translational journey. Trends Mol Med.

[105] Han Y, Duan X, Yang L, Nilsson-Payant BE, Wang P, Duan F, et al. Identification of SARS-CoV-2 inhibitors using lung and colonic organoids. Nature. 2020;589(7841):270–75. 10.1038/s41586-020-2901-9 PMC8034380 33116299

[106] Zhao B, Ni C, Gao R, Wang Y, Yang L, Wei J, et al. Recapitulation of SARS-CoV-2 infection and cholangiocyte damage with human liver ductal organoids. Protein Cell. 2020;11(10):771–5. 10.1007/s13238-020-00718-6 PMC7164704 32303993

[107] Monteil V, Kwon H, Prado P, Hagelkruys A, Wimmer RA, Stahl M (2020). Inhibition of SARS-CoV-2 infections in engineered human tissues using clinical-grade soluble human ACE2. Cell..

[108] Rajan SAP, Aleman J, Wan M, Pourhabibi Zarandi N, Nzou G, Murphy S (2020). Probing prodrug metabolism and reciprocal toxicity with an integrated and humanized multi-tissue organ-on-a-chip platform. Acta Biomater.

[109] Meyer-Berg H, Zhou Yang L, Pilar de Lucas M, Zambrano A, Hyde SC, Gill DR (2020). Identification of aav serotypes for lung gene therapy in human embryonic stem cell-derived lung organoids. Stem Cell Res Ther.

[110] Katsura H, Sontake V, Tata A, Kobayashi Y, Edwards CE, Heaton BE, et al. Human lung stem cell-based alveolospheres provide insights into SARS-CoV-2-mediated interferon responses and pneumocyte dysfunction. Cell Stem Cell. 2020;27(6):890–904. 10.1016/j.stem.2020.10.005 PMC7577733 33128895

[111] Atala A, Henn A, Lundberg M, Ahsan T, Greenberg J, Krukin J, et al. Regen med therapeutic opportunities for fighting covid-19. Stem Cells Transl Med. 2020;10(1):5–13. 10.1002/sctm.20-0245 PMC7461298 32856432

[112] Johansen MD, Irving A, Montagutelli X, Tate MD, Rudloff I, Nold MF (2020). Animal and translational models of SARS-CoV-2 infection and covid-19. Mucosal Immunol.

